# Physical performances show conflicting associations in aged manual workers

**DOI:** 10.1038/s41598-020-59050-y

**Published:** 2020-02-10

**Authors:** Kristoffer L. Norheim, Afshin Samani, Jakob H. Bønløkke, Øyvind Omland, Pascal Madeleine

**Affiliations:** 1 Sport Sciences - Performance and Technology, Department of Health Science and Technology, Faculty of Medicine, Aalborg University, Aalborg, Denmark; 2 Department of Occupational and Environmental Medicine, Danish Ramazzini Centre, Aalborg University Hospital, Aalborg, Denmark

**Keywords:** Ageing, Occupational health

## Abstract

Ageing is associated with a decrease in physical performance implying that aged manual workers may be unable to match the physical requirements of their jobs. In this cross-sectional study, 96 male manual workers aged 51–72 years were recruited. Outcomes included handgrip strength (HGS), fat-free mass (FFM), fat percentage, cardiorespiratory fitness ($\dot{{\rm{V}}}$O_2_max), forced vital capacity (FVC), forced expiratory volume after 1 s (FEV_1_), spinal flexibility, sit-to-stand test performance and static balance. Covariates included height, smoking habits, leisure-time physical activity and systemic inflammation from blood samples. Outcomes were also compared with general populations. Age was negatively related to FFM and FEV_1_, whereas static balance (velocity of displacement) was positively associated with age. Greater HGS, but poorer $\dot{{\rm{V}}}$O_2_max and FEV_1_/FEV ratio were found compared with general populations. Age was negatively related with physical performances although a large part of the variance in performance could be explained by factors other than age such as smoking and systemic inflammation. The manual workers had greater muscle strength but had poorer cardiorespiratory fitness and lung function when compared with general populations. Specific health interventions targeting specifically cardiorespiratory fitness, lung function, and balance may be needed to maintain physical performances among manual workers.

## Introduction

Noticeable age-related reductions in physical performance typically start beyond the third^1^ and accelerate after the fifth decade of life^2–5^. These changes include reduced muscle strength^3^ and cardiorespiratory fitness^4^, altered body composition^2^ and impaired lung function^4^. Together, these adverse effects of ageing eventually culminate in diminished physical performances and mobility impairments^6,7^. An inactive life style has been ascribed as an important factor contributing to such changes and increasing physical activity–through either aerobic or resistance-based exercise–may mitigate some of the negative effects of age on physical performances^6,8^.

Adults who have physically demanding jobs are inherently physically active in their working life. Intuitively, this should retain their physical performance to a greater extent than that of people doing sedentary work^8–10^. However, the physical activities performed by manual workers may help in maintaining muscle strength^11^, but do not seem to improve cardiorespiratory fitness^11–15^. Moreover, a recent systematic review has shown that high occupational physical activity increases the risk of all-cause mortality by 18%^16^. Average life expectancy has steadily increased over the last century, which has led policymakers to pass laws that increase retirement age in an attempt to accommodate the growing population of senior workers. The effect of increasing retirement age may therefore be to shorten the years lived in good health for manual workers^17^. Although muscle strength is reportedly higher in manual workers when compared with the general population^11^, the ageing process still takes place^18^. Indeed, long-term exposure to strenuous work without adequate rest may cause physical degeneration, especially when combined with low-grade inflammation due to adiposity and tobacco use, thus rendering them more vulnerable to musculoskeletal and cardiovascular disorders^17,19^. A better understanding of the age-related differences in physical performance among manual workers might shed light on physical deficiencies that could be targeted for interventions possibly contributing to better work capability as well as a meaningful retirement for all workers^20,21^. However, the use of a control group not having been exposed to the same physical demands is generally an issue due to e.g. different working life history, various levels of work experience, and the presence of musculoskeletal pain^22,23^. One way to circumvent that is to compare the studied participants with existing cohorts^3^.

Thus, the main objectives of this cross-sectional study were (i) to investigate age-related differences in physical performances among Danish manual workers in their last two decades of working life and (ii) to compare physical performances of these Danish manual workers with general populations. We hypothesized that physical performance would be negatively associated with age and that manual workers would be stronger but have similar or worse cardiorespiratory fitness when compared with the general population. This study was conducted on a sub-sample of older manual workers that was part of an ongoing cohort study. The comparisons are made with general populations from Denmark, Great Britain, Norway, Switzerland, and United States that most likely also included some manual workers.

## Methods

### Study recruitment and design

This study is a part of a larger project that concern senior Danish manual workers from the ALFA-cohort (ALdring og Fysisk Arbejde; Ageing and Physical Work). The project included a register-based cohort of manual workers where in 2016 a random sample of 2727 workers aged 50 to 70 years responded to an online or postal-based questionnaire assessing work environment and health. The workers who reported interest in participating in a laboratory examination were contacted through e-mail and telephone when available (n = 605). In line with the conducted power calculation^24^, 97 ethnically Danish participants (96 males, 1 female) underwent laboratory assessments of physical performances. These assessments were conducted approximately one and a half year after participants had answered the ALFA questionnaire and they were therefore aged 51–72 years at this time. Due to the uneven gender distribution, only the male participants were included in the present analyses. All participants gave written and oral informed consent to participate after being informed about the purpose of the study. The study follows STROBE guidelines and was carried out in accordance to the Helsinki declaration and approved by The North Denmark Region Committee on Health Research Ethics (N-20160023). To increase the feasibility of the study, participants arrived non-fasting in the laboratory in either the morning or early afternoon depending on their working schedule. The protocol for this study and test-order has been described in detail previously^24^. Inter-test fatigue was assessed using the Borg scale^25^.

### Systemic inflammation

Prior to all physical tests, non-fasting venous blood samples were drawn into 6-mL EDTA tubes and centrifuged for 10 min at 2000 g at 4 °C. Plasma was stored at −80 °C until analysed. Enzyme-linked immunosorbent assay kits from R&D Systems (Minneapolis, MN, USA) were used to measure plasma concentrations of C-reactive protein (Human CRP Quantikine ELISA Kit DCRP00) and interleukin-6 (Human IL-6 Quantikine HS ELISA Kit HS600B). All samples were measured in duplicates, with a mean intra-assay variation of 1.08% for C-reactive protein (CRP) and 8.75% for interleukin-6 (IL-6), and mean concentrations were used for analyses. The limit of detection was 0.010 µg·L^−1^ for CRP and 0.039 ng·L^−1^ for IL-6. Samples from 19 of the 97 included subjects could not be attained due to unforeseen technical difficulties including periodically absence of laboratory support.

### Body composition

Body composition was estimated using bioelectrical impedance analysis (InBody 370, Biospace, Seoul, Korea) following the manufacturer’s recommendations. The outcomes included fat-free mass (FFM) and fat percentage.

### Lung function

Lung function was measured using a Spirobank II® SMART (Medical International Research, Rome, Italy) spirometer. In line with^26^, participants completed 3–8 forced maximal expirations in a standing position with a nose clip. The main outcome metrics were forced expiratory volume after 1 s (FEV_1_), forced vital capacity (FVC) and the FEV_1_/FVC ratio.

### Muscle strength

Maximal isometric handgrip strength (HGS) was measured using a digital dynamometer (Model G100, Biometrics Ltd, Gwent, UK). Briefly, participants sat in a chair holding the dynamometer in their dominant hand with their lower arm resting on an armchair^27^. Three maximal trials lasting 3 s separated by 2 min rest were completed and the highest value was defined as maximal HGS.

### Physical function

Flexibility of the spine and pelvis was assessed using the fingertip-to-floor test^28^, where lower values (in cm) expresses greater flexibility. A 5-repetition sit-to-stand test (STS) was used to assess lower extremity function^29^ and total completion time was noted in s. Static balance was assessed using a force platform (AMTI AccuSway, Watertown, MA, USA) during 60-s of quiet standing with both eyes open and eyes closed. Static balance (balance) was expressed as the total mean velocity of displacement of the centre of pressure with eyes closed since velocity measures during eyes closed conditions seem most sensitive in detecting age-related differences^30^. Five out of the 97 included participants did not complete the balance test due to technical issues.

### Cardiorespiratory fitness

Cardiorespiratory fitness, expressed as an estimation of the maximal rate of oxygen consumption ($\dot{{\rm{V}}}$O_2_max), was tested using a bicycle ergometer (Ergomedic 874E, Monark AB, Varberg, Sweden) as described previously^24^. The computer solution for the Åstrand-Ryhming nomogram^31^ was used to calculate estimated $\dot{{\rm{V}}}$O_2_max^32^ (see Supplementary Methods), which was expressed both in absolute (a$\dot{{\rm{V}}}$O_2_max: L·min^−1^) and relative (r$\dot{{\rm{V}}}$O_2_max: ml·kg^−1^·min^−1^) terms. Nineteen out of the 97 included participants did not complete the cycling test. This was due to some participants not being able to reach a sufficient HR (i.e. >120 bpm) before feeling exhausted (n = 6) or having knee pain (n = 5) as well as technical issues with the ergometer (n = 8).

### Self-reported indices

A short version of the ALFA-questionnaire was answered during the laboratory assessments^24^. Leisure-time physical activity (LTPA) was assessed in accordance with^33^. Briefly, hours per week doing low, moderate and high intensity exercise were converted into metabolic equivalents (METs). In accordance with recommendations set by the American College of Sports Medicine (ACSM), participants were classified into either Low, Moderate or High^34^. Smoking status was noted as current, former or never smoker. Self-reported diseases were also noted.

### Population comparisons

Data on pulmonary function were compared with the most recent Danish reference values^35^ and the number of participants with values below the lower limit of normal (LLN) was calculated. To allow for direct comparisons of biometric and physical performance outcomes with values from general populations, reference values in age strata of 50–59 and 60–69 years were obtained when available. Danish reference values for handgrip strength were obtained from the Copenhagen Sarcopenia Study^36^. Data from the National Health and Nutrition Examination Survey (NHANES)^37^ was obtained to represent the general population of the United States. Only data from non-Hispanic white males was extracted. When available, comparisons were also made with published values from Norway^4^, Switzerland^2^, and Great Britain^3^. It is important to note that the compared populations most likely also contained a proportion of manual workers. This may actually result in an underestimation of the observed differences.

### Statistics

All continuous data was tested for normality using the Shapiro-Wilk test. CRP and IL-6 data was non-normally distributed and therefore log transformed. Simple associations between age and the dependent variables HGS, FFM, fat percent, a$\dot{{\rm{V}}}$O_2_max, r$\dot{{\rm{V}}}$O_2_max, FEV_1_, FVC, FEV_1_/FVC, flexibility, STS and balance was assessed using linear regression (Model 1). To estimate relative changes with age, these outcomes were log transformed using the natural logarithm and multiplied by 100; thus, β-coefficients and 95% confidence intervals (CI) of the independent variables represent symmetric percentage units of the dependent variable, as suggested by Cole^38^. Multiple linear regression models were created by entering height and smoking (1 = current, 0 = former or never) (Model 2), and additionally LTPA (1 = Low, 0 = Moderate or High), log CRP, and log IL-6 into the models (Model 3). To measure the proportion of variance in the outcomes explained by adding each independent variable, while taking into account the relationships among all the variables, the squared semi-partial correlation coefficient (∆R2) was calculated for Model 3^39^. Comparisons between biometric and physical performance outcomes from the present study and available values from general populations were made using z-scores to indicate the number of standard errors between the sample and population means and effect size was estimated as Cohen’s d. Statistical significance was set to *P* < 0.05 and analyses were carried out using SPSS 25.0 (SPSS Inc., Chicago, Illinois, USA).

## Results

### Characteristics and generalisability of the assessed population

Results from the 2016 ALFA questionnaire indicated that the participants recruited for laboratory assessments and the remaining questionnaire respondents were comparable except for a greater proportion of bricklayers (23.6% vs. 9.9%, *p* < 0.001) in the present study (see Supplementary Table S1). Of the 96 included male manual workers, a large proportion reported a moderate level of leisure-time physical activity and were previous smokers (Table 1). The most common self-reported diseases were arthritis and hypertension. Inter-test ratings of perceived exertion (RPE) indicated no carryover fatigue from other tests prior to the balance test, RPE 7.6 ± 1.5, the handgrip test, RPE 8.8 ± 1.6, nor the cycling test, RPE 11.8 ± 1.9. The total test-battery was rated as light, RPE 10.8 ± 1.5 (11 = “fairly light”).

**Table 1:** Characteristics of participants.

	n = 96
Age (year)	60.5 ± 5.8
Height (cm)	176.9 ± 6.7
Body mass (kg)	88.0 ± 13.0
Body mass index (kg m^−2^)	28.0 ± 3.3
Smoking (%)	
Never	28.1
Previous	51.1
Current	20.8
Leisure-time physical activity (%)	
Low	10.4
Moderate	76.0
High	13.6
Self-reported diseases (%)	
COPD	4.2
Asthma	5.2
Other lung diseases	2.1
Hypertension	22.9
Arthritis	26.0
Diabetes mellitus	5.2

Values are mean ± standard deviation or percent (%). COPD, chronic obstructive pulmonary disease.

### Physical performance and age

In the simple linear regression models, age was negatively related to HGS, FFM, a$\dot{{\rm{V}}}$O_2_max, FEV_1_ and FVC, whereas balance was positively related to age. Of these, the estimated yearly percent change in physical performance was smallest for FVC and greatest for balance (Table 2). In the fully adjusted models, age explained a mean additional 3.2 ± 4.0% of the variance in the physical performance outcomes and remained a significant independent predictor of FFM, FEV_1_ and balance (see Supplementary Table S2). Notably, CRP explained a mean additional 3.8 ± 3.5% of the variance in performance, whereas smoking and IL-6 explained an additional 2.0 ± 2.3% and 1.3 ± 1.8%, respectively.

**Table 2:** Symmetric percentage changes in biometric and physical performance outcomes based on age in years.

	Model 1			Model 2^a^			Model 3^b^		
	β	95% CI	n	β	95% CI	n	β	95% CI	n
HGS	**−0.89**	**−**1.50 to −0.29	96	**−**0.42	**−**1.00 to 0.17	96	**−**0.44	**−**1.11 to 0.23	77
FFM	**−0.94**	**−**1.40 to −0.48	96	**−0.33**	**−**0.61 to −0.05	96	**−0.39**	**−**0.71 to −0.08	77
Fat percent	0.25	**−**0.58 to 1.08	96	**−**0.13	**−**0.98 to 0.72	96	**−**0.01	**−**0.87 to 0.85	77
a$\dot{{\rm{V}}}$O_2_max	**−1.14**	**−**2.15 to **−**0.13	77	**−1.12**	**−**2.12 to **−**0.12	77	**−**0.88	**−**2.01 to 0.26	62
r$\dot{{\rm{V}}}$O_2_max	**−**0.30	**−**1.36 to 0.77	77	**−**0.61	**−**1.72 to 0.51	77	**−**0.44	**−**1.68 to 0.80	62
FEV_1_	**−1.26**	**−**2.13 to **−**0.39	96	**−**0.65	**−**1.44 to 0.14	96	**−0.86**	**−**1.64 to **−**0.07	77
FVC	**−0.86**	**−**1.49 to **−**0.22	96	**−**0.30	**−**0.83 to 0.24	96	**−**0.36	**−**0.94 to 0.22	77
FEV_1_/FVC	**−**0.40	**−**0.90 to 0.10	96	**−**0.34	**−**0.87 to 0.18	96	**−**0.48	**−**1.04 to 0.07	77
Flexibility	**−**0.21	**−**1.83 to 1.41	96	0.10	**−**1.59 to 1.80	96	0.85	**−**0.80 to 2.50	77
STS	0.22	**−**0.56 to 1.01	96	0.36	**−**0.48 to 1.19	96	0.37	**−**0.48 to 1.26	77
Balance	**1.90**	0.53 to 3.27	91	**2.48**	1.09 to 3.87	91	**2.73**	1.20 to 4.27	72

Note that the dependent variables are 100 ln so that coefficients represent the symmetric percentage change in the dependent variable per yearly increase in age. Significant β-coefficients are indicated in bold font when *p* < 0.05.

CI, confidence interval; HGS, handgrip strength; FFM, fat-free mass; a$\dot{{\rm{V}}}$O_2_max, absolute maximal rate of oxygen uptake; r$\dot{{\rm{V}}}$O_2_max, relative maximal rate of oxygen uptake; FEV_1_, forced expiratory volume after 1 s; FVC, forced vital capacity; STS, sit-to-stand test.

^a^Adjusted for height and smoking (current).

^b^Adjusted for height, smoking (current), leisure-time physical activity (Low), Log C-reactive protein, and Log interleukin-6.

### Population comparisons

Based on reference equations on lung function among the Danish population, the percent predicted values for FEV_1_, FVC, and FEV_1_/FVC were 93.8 ± 17.9, 104.6 ± 15.1 and 90.1 ± 11.4, respectively. Consequently, 14, 3 and 29 participants had values below the LLN for FEV_1_, FVC, and FEV_1_/FVC, respectively. For the comparisons in Table 3, seven participants aged 70+ years were excluded in the comparison of measured values with values from general populations to accommodate the available reference frames. Body mass index (BMI) was significantly greater for both the 50–59 and 60–69 years age groups in the present study when compared with values from both Norway and Switzerland, but not compared with the United States. HGS was higher only for the 60–69 years age group when compared with Danish reference values. HGS was higher for both age groups compared with both the United States and Great Britain. FFM was higher for the 50–59 years group in the present study compared with the United States, whereas compared with the Swiss population both FFM and fat percent were significantly higher in the present study for both age groups. Poorer cardiorespiratory fitness was found compared with Norwegians. Lung function was similar between populations except for FEV_1_/FVC, which was also lower in the present study when compared with the Norwegian population for both age groups.

**Table 3:** Comparison of biometric and physical performance values from general populations.

Variable	Age	Present study	General populations														
			Denmark^36^			United States^37^			Norway^4^			Switzerland^2^			Great Britain^3^		
		Mean	Mean	Z-score	Effect size*	Mean	Z-score	Effect size	Mean	Z-score	Effect size	Mean	Z-score	Effect size	Mean	Z-score	Effect size
BMI (kg·m^−2^)	50–59	28.2				27.8	0.58	0.10	26.8	**3.44**	0.50	24.9	**7.55**	1.13			
BMI (kg·m^−2^)	60–69	28.1				27.6	0.80	0.13	26.9	**2.30**	0.33	25.3	**6.26**	0.83			
HGS (kg)	50–59	52.5	50.3	1.91	0.28	45.9	**5.42**	0.82							46.2	**4.25**	0.70
HGS (kg)	60–69	51.0	47.5	**2.82**	0.41	42.9	**6.92**	0.97							42.3	**6.81**	1.00
FFM (kg)	50–59	68.1				64.6	**2.65**	0.40				58.6	**9.57**	1.20			
FFM (kg)	60–69	63.4				62.3	0.83	0.14				57.1	**6.40**	0.92			
Fat percent (%)	50–59	24.8				25.1	−0.29	0.05				21.7	**4.01**	0.68			
Fat percent (%)	60–69	26.4				26.2	0.22	0.03				22.0	**5.76**	0.62			
$\dot{{\rm{V}}}$O_2_max (L·min^−1^)	50–59	2.52							3.14	−**8.39**	1.13						
$\dot{{\rm{V}}}$O_2_max (L·min^−1^)	60–69	2.19							2.74	−**7.69**	1.11						
$\dot{{\rm{V}}}$O_2_max (ml·kg·min^−1^)	50–59	28.2							36.8	−**8.64**	1.24						
$\dot{{\rm{V}}}$O_2_max (ml·kg·min^−1^)	60–69	25.8							32.4	−**6.92**	1.07						
FEV_1_ (L)	50–59	3.56				3.49	0.71	0.12	3.70	−1.86	0.23						
FEV_1_ (L)	60–69	3.29				3.12	1.63	0.26	3.40	−1.23	0.18						
FVC (L)	50–59	4.90				4.76	1.15	0.20	5.00	−0.95	0.15						
FVC (L)	60–69	4.62				4.40	1.68	0.27	4.60	0.17	0.03						
FEV_1_/FVC (%)	50–59	72.4				73.3	−0.71	0.10	75.8	−**3.44**	0.40						
FEV_1_/FVC (%)	60–69	70.8				70.8	0.00	0.00	73.7	−**2.62**	0.38						

Significant z-scores are indicated in bold font when p < 0.05.

BMI, body mass index; HGS, handgrip strength; FFM, fat-free mass; $\dot{{\rm{V}}}$O_2_max, maximal rate of oxygen uptake; FEV_1_, forced expiratory volume after 1 s; FVC, forced vital capacity.

*Cohen’s d.

## Discussion

This study examined age-related differences in physical performance among adults in their last two decades of working life. The recruited participants were representative of male Danish manual workers in their sixth and seventh decade of life. In line with our hypothesis, age was negatively related with physical performances although a large part of the variance in performance could be explained by factors other than age such as smoking and systemic inflammation. Further, we showed that Danish manual workers had greater muscle strength but had poorer cardiorespiratory fitness and lung function when compared with general populations.

Age-related reductions in muscle strength are reported to be 2–5 times faster than that of muscle mass^40^. For example, in a cross-sectional study of males aged 45 to 78 years the estimated mean annual losses of muscle strength and FFM were −1.1% and −0.4%, respectively^41^. In older cohorts (65–89 years), estimated changes of −1.7% per year have been found for handgrip strength^42^. In our fully adjusted regression models, the estimated changes in HGS and FFM mass among male manual workers were both −0.4% per year. These findings show both less loss of muscle strength than in previous studies and a decline in FFM comparable with previous studies. Manual workers thus seem to retain more of the muscle strength relative to their muscle mass compared with the general population. Regarding cardiorespiratory fitness, annual reductions in $\dot{{\rm{V}}}$O_2_max of −1.0% per year have been reported^43^, which is comparable to that we found in the present study. However, only absolute and not relative $\dot{{\rm{V}}}$O_2_max was related with age thus arguing that this association was driven primarily through age-related changes in body size. After the age of 30 years, FEV_1_ and FVC have been shown to decline at a rate of about −0.8%/year and −0.6%/year in the Dutch population^5^. With comparable proportions of smokers between studies, our findings were similar to the Dutch study albeit with a slightly greater estimated decline in the simple regression models. Although tobacco smoking may increase systemic inflammation, elevated levels of CRP are associated with lung function decline independent of smoking status^44^, which was supported by the present study. Indeed, CRP explained a greater portion of the variance in FEV_1_ than to smoking status (4.7% vs. 0.5%). This highlights the limitation of using self-reported measurements such as smoking status while strengthening the use of CRP as a biomarker of physical performance. Elevated CRP levels are also linked to chronic diseases, e.g. to cardiovascular disease and musculoskeletal disorders such as arthritis^45^. Even though aerobic exercise training has been shown to decrease CRP among manual workers^46^, this effect seems primarily to be mediated through changes in body fat^47^ which is in line with CRP explaining 11.1% of the variance in body fat percent. Also in agreement with our findings, balance expressed as the total mean velocity of centre of pressure displacement has been reported to increase by approximately 2.0% per year^30^, indicating increased instability with age. Postural instability poses a particular risk for adults exposed to awkward postures and physically demanding work-tasks such as manual workers by increasing the risk of accidental falls. Thus, physical activities challenging balance could be implemented to decrease fall risks^9^. Finally, no significant changes were found for fat percent, back flexibility and STS test performance.

The included male manual workers had greater HGS, FFM, and fat percentage, but poorer cardiorespiratory fitness and lung function when compared with general populations. Although populations were comparable in terms of gender, age and ethnicity, a greater absolute body size may have primarily driven differences found between the Norwegian and Swiss population. Nonetheless, r$\dot{{\rm{V}}}$O_2_max and FEV_1_/FVC ratio were significantly poorer in the present study indicating differences regardless of body size. Moreover, twenty-nine of the participants had FEV_1_/FVC below the LLN for Danish males. This suggests that manual workers would benefit from respiratory muscle training^48^ and aerobic conditioning^6^. Indeed, both absolute and relative cardiorespiratory fitness were significantly lower for both the 50–59 and 60–69 years age groups when compared with the Norwegian population. This finding should however be interpreted with caution as the present study estimated $\dot{{\rm{V}}}$O_2_max based on a sub-maximal test, whereas a maximal test measuring gas exchanges was used in^3^. Even so, similar values to the ones we measured have been found among fire fighters based on direct measurements of $\dot{{\rm{V}}}$O_2_max^49^. Thus, either the manual workers in our study had poorer cardiorespiratory health since youth (compared with the Norwegian general population) or there does not seem to be any cardiorespiratory training or maintaining effect of manual work in agreement with^12–15^. As such, low cardiorespiratory fitness increases the relative load when performing a given task and when combined with heavy physical strain at work it has been related to an increased risk of disability pension^19^. Concerning body composition, FFM was significantly higher for the 50–59 years age group when compared with the United States population (both populations had similar body size) and the difference in FFM per decade was 2.3 kg and 4.7 kg for the present study and the US population, respectively. This could indicate a greater decline in FFM among male manual workers than that seen in the United States although the estimated changes in FFM with age were similar to other studies. Similar to other findings^50^, HGS was greater in the present study compared with both the United States and Great Britain general population. Compared with the Danish population, HGS was only significantly greater for the 60–69 years age group, which could indicate that manual work preserved muscle strength. Taken together, the results of the present study show that handgrip force was greater than that seen in general populations highlighting that physical work preserves strength. Paradoxically, cardiorespiratory fitness and lung function were poorer and decreased at similar rates in manual workers as those seen in other studies^5,43^. These findings solidify further the health paradox stating that occupational and leisure-time physical activity cause opposing effects^10,16,51^. Our findings also argue for the simultaneous use of measures describing both musculoskeletal, cardiorespiratory, and pulmonary capabilities to better understand age-related differences in physical performance.

As outlined in^17^, deterioration of physical performance among workers poses a serious problem not only for the individual but also for society as a whole. The findings of the present study indicate that physical performance among male manual workers in their last twenty years of working were both increased and reduced in terms of handgrip strength and cardiorespiratory fitness or lung function. Handgrip strength was higher while $\dot{{\rm{V}}}$O_2_max and FEV_1_/FVC ratio were poorer than general populations. Future studies should therefore focus on maintaining or increasing cardiorespiratory fitness and lung function in these workers. Specific health interventions targeting cardiorespiratory fitness, lung function, and balance may be needed to maintain physical capability among manual workers^6^. Moreover, promotions to reduce unhealthy behaviours such as smoking and unhealthy diets may improve lung function, body composition, and general health through fat loss and mitigation of systemic inflammation.

One of the limitations of the present study is its cross-sectional design which could be subject to cohort bias and an underestimation of changes in physical performance with age. We moreover acknowledge that cause-effect relationships cannot be inferred from cross-sectional studies. The reported findings moreover need to be interpreted with caution considering that the general populations used as comparisons probably contained a certain proportion of manual workers potentially washing out differences between populations. However, selecting a control group with no experience of manual work at all would have been challenging^22,23^. Still the present study is unique in its kind since it combines a vast numbers of biological biomarkers used to anchor the effects of the last two decades of work in manual workers. Further, our studied sample (only composed of Danish males) may limit generalisability to other groups. However, except for a larger percentage of bricklayers, the general characteristics of the included participants were similar to the random sample of 2630 workers who only answered the questionnaire thus indicating that our findings can at least be extrapolated to male Danish manual workers aged 50–70 years. Finally, future studies assessing the effects of ageing on physical performances should also address comorbidity issues^52,53^.

In conclusion, age was negatively related with physical performances although a large part of the variance in performance could be explained by factors other than age such as smoking and systemic inflammation. Manual workers had greater muscle strength but had poorer cardiorespiratory fitness and lung function when compared with general populations.

## Supplementary information

Supplementart information.

## Data Availability

The datasets generated during and/or analysed during the current study are available from the corresponding author on reasonable request.
