# Supplementary material for: Physical performances show conflicting associations in aged manual workers
Source: Sci Rep. 2020 Feb 10;10:2254. doi: 10.1038/s41598-020-59050-y (PMC7010773; doi:10.1038/s41598-020-59050-y)
Supplement: Supplementary file 1 — Supplementart information. [file 41598_2020_59050_MOESM1_ESM.pdf]

Physical performances show conflicting associations in aged manual workers

Kristoffer L. Norheim, Afshin Samani, Jakob Hjort Bønløkke, Øyvind Omland, Pascal Madeleine

Supplementary Methods. The computer solution for the Åstrand-Ryhming nomogram used to calculate estimated $\dot{V}O_2max$

First, body surface area (BSA) is estimated [1]

$$BSA(m^2) = M^{0.425} \times H^{0.725} \times 71.84 \times 10^{-4} \quad [1]$$

where M is body mass in kilograms and H is standing height in centimeters. The submaximal rate of oxygen consumption is estimated as [2]

$$\dot{V}o_2 = \frac{(W \times 62.28) + (670 \times BSA)}{5} \quad [2]$$

where W is the steady-state load during cycling (watt). This formula is based on three assumptions: bicycle ergometer exercise has an efficiency of approximately 23%; 5 calories are burned for each liter of oxygen consumed, and; basal energy expenditure can be estimated from BSA. The Åstrand-Ryhming nomogram estimates $\dot{V}O_2max$ based on a sex-specific formula for men [3]

$$\dot{V}o_{2max} = \frac{(195 - 61)}{(P - 61)} \times Vo_2 \quad [3]$$

where P is the steady-state HR (bpm; beats per min) during exercise and should lie in the range of 122-172 bpm for men. Lastly, $\dot{V}_{O2max}$ values are age-corrected by the sex-specific formula for men [4]

$$Correction_{male} = \frac{100}{100 + [1.37(Age) - 33.2]} \quad [4]$$

where age is chronological age in whole years.

Supplementary Table S1. Participants' characteristics of the Ageing and Physical Work (ALFA) cohort answering to the questionnaire and of the volunteers taking part in the clinical assessments.

	ALFA cohort (n=2630)		Clinical population (n=97)	
	Mean or %	95% CI	Mean or %	95% CI
Gender (women)	2.0		1.0	
Age (year)	59.6	59.3 to 59.8	59.5	58.4 to 60.7
Height (m)	1.79	1.79 to 1.79	1.78	1.77 to 1.79
Body mass (kg)	87.2	86.6 to 87.8	85.8	83.3 to 88.4
BMI (kg·m^{-2})	27.6	26.8 to 28.4	27.1	26.5 to 27.7
Work experience (year)	31.1	30.5 to 31.7	29.1	25.9 to 32.4
Physical work ability (%)				
Poor	5.0		2.8	
Fair	25.2		23.6	
Good	40.7		45.8	
Very good	23.3		19.4	
Excellent	5.8		8.3	
Profession				
Carpenter	22.1		22.2	
Electrician	12.4		6.9	
Bricklayer	9.9		23.6*	
Plumber	6.6		8.3	
Painter	6.0		2.8	
Other	43.1		36.1	
Leisure-time physical activity (%)				
Low	14.9		11.5	
Moderate	73.3		72.4	
High	11.8		16.1	
Smoking status (%)				
Never	37.7		29.5	
Previous	38.6		50.5	
Current	23.7		20.0	

*Significantly larger proportion ($p < 0.001$).

Supplementary Table S2. Symmetric percentage changes in biometric and physical performance outcomes based on age in years.

		Model 1		Model 2		Model 3		
DV	IV	β	95% CI	β	95% CI	β	95% CI	ΔR^2
HGS	Age (year)	**-0.89**	-1.50 to -0.29	-0.42	-1.00 to 0.17	-0.44	-1.11 to 0.23	1.69
	Height (cm)			**1.22**	0.72 to 1.72	**1.25**	0.67 to 1.80	19.62
	Smoking (current)			2.71	-5.01 to 10.49	5.03	-5.25 to 15.3	0.94
	LTPA (Low)					-9.93	-23.0 to 3.13	2.28
	Log CRP					-5.54	-15.9 to 4.80	1.12
	Log IL-6					-1.95	-18.3 to 14.4	0.06
		N = 96, aR^2 = 0.07		N = 96, aR^2 = 0.25		N = 77, aR^2 = 0.25		
FFM	Age (year)	**-0.94**	-1.40 to -0.48	**-0.33**	-0.61 to -0.05	**-0.39**	-0.71 to -0.08	2.19
	Height (cm)			**1.64**	1.40 to 1.88	**1.66**	1.39 to 1.92	57.00
	Smoking (current)			**-5.09**	-8.82 to -1.35	**-5.22**	-10.1 to -0.37	1.66
	LTPA (Low)					**-8.38**	-14.5 to -2.23	2.66
	Log CRP					-1.65	-6.53 to 3.23	0.16
	Log IL-6					0.30	-7.39 to 7.99	< 0.01
		N = 96, aR^2 = 0.14		N = 96, aR^2 = 0.72		N = 77, aR^2 = 0.73		
Fat percent	Age (year)	0.25	-0.58 to 1.08	-0.13	-0.98 to 0.72	-0.01	-0.87 to 0.85	< 0.01
	Height (cm)			**-1.05**	-1.79 to -0.32	**-1.04**	-1.75 to -0.32	8.18
	Smoking (current)			5.50	-5.89 to 16.9	-4.74	-17.9 to 8.41	0.50
	LTPA (Low)					-5.84	-22.5 to 10.9	0.48
	Log CRP					**31.6**	18.3 to 44.8	11.09
	Log IL-6					-4.67	-25.5 to 16.2	0.19
		N = 96, aR^2 < 0.01		N = 96, aR^2 = 0.07		N = 77, aR^2 = 0.26		
$a\dot{V}O_{2max}$	Age (year)	**-1.14**	-2.15 to -0.13	**-1.12**	-2.12 to -0.12	-0.88	-2.01 to 0.26	2.99
	Height (cm)			**1.27**	0.42 to 2.13	**1.44**	0.50 to 2.38	11.70
	Smoking (current)			**-24.0**	-42.3 to -5.71	-22.1	-45.9 to 1.67	4.33
	LTPA (Low)					-7.14	-29.9 to 15.6	0.49
	Log CRP					-14.2	-31.4 to 3.10	3.39

	Log IL-6					-23.9	-52.0 to 4.23	3.61
			N = 77, aR^2 = 0.05		N = 77, aR^2 = 0.18		N = 62, aR^2 = 0.24	
$r\dot{V}O_{2max}$	Age (year)	-0.30	-1.36 to 0.77	-0.61	-1.72 to 0.51	-0.44	-1.68 to 0.80	0.72
	Height (cm)			-0.10	-1.06 to 0.86	0.14	-0.89 to 1.16	0.10
	Smoking (current)			-19.3	-39.7 to 1.13	-19.0	-44.9 to 6.91	0.03
	LTPA (Low)					1.63	-23.1 to 26.3	2.28
	Log CRP					**-22.3**	-41.1 to -3.50	1.12
	Log IL-6					-19.7	-50.3 to 10.8	0.06
			N = 77, aR^2 < 0.01		N = 77, aR^2 = 0.01		N = 62, aR^2 = 0.13	
FEV_1	Age (year)	**-1.26**	-2.13 to -0.39	-0.65	-1.44 to 0.14	**-0.86**	-1.64 to -0.07	3.13
	Height (cm)			**1.75**	1.07 to 2.43	**1.86**	1.21 to 2.50	21.43
	Smoking (current)			**-16.2**	-26.8 to -5.67	-5.02	-17.0 to 6.94	0.46
	LTPA (Low)					**-28.6**	-43.8 to -13.4	9.30
	Log CRP					**-16.1**	-28.2 to -4.09	4.71
	Log IL-6					-6.54	-25.5 to 12.4	0.31
			N = 96, aR^2 = 0.07		N = 96, aR^2 = 0.33		N = 77, aR^2 = 0.50	
FVC	Age (year)	**-0.86**	-1.49 to -0.22	-0.30	-0.83 to 0.24	-0.36	-0.94 to 0.22	1.06
	Height (cm)			**1.57**	1.10 to 2.03	**1.51**	1.04 to 1.99	27.67
	Smoking (current)			**-10.4**	-17.6 to -3.24	-5.33	-14.1 to 3.48	1.00
	LTPA (Low)					**-17.6**	28.7 to -6.38	6.76
	Log CRP					-8.54	-17.4 to 0.32	2.56
	Log IL-6					-4.69	-18.7 to 9.28	0.31
			N = 96, aR^2 = 0.06		N = 96, aR^2 = 0.40		N = 77, aR^2 = 0.48	
FEV_1/FVC	Age (year)	-0.40	-0.90 to 0.10	-0.34	-0.87 to 0.18	-0.48	-1.04 to 0.07	3.50
	Height (cm)			0.19	-0.26 to 0.64	0.35	-0.11 to 0.81	2.62
	Smoking (current)			-5.60	-12.6 to 1.42	0.28	-8.19 to 8.75	< 0.01
	LTPA (Low)					**-10.8**	-21.6 to -0.08	4.67
	Log CRP					-7.39	-15.9 to 1.13	3.46
	Log IL-6					-1.47	-14.9 to 12.0	0.06
			N = 96, aR^2 = 0.01		N = 96, aR^2 = 0.03		N = 77, aR^2 = 0.12	

Flexibility	Age (year)	-0.21	-1.83 to 1.41	0.10	-1.59 to 1.80	0.85	-0.80 to 2.50	1.30
	Height (cm)			0.57	-0.90 to 2.04	0.51	-0.86 to 1.88	0.69
	Smoking (current)			**24.6**	1.92 to 47.4	22.8	-2.47 to 48.0	4.00
	LTPA (Low)					25.4	-6.68 to 57.4	3.06
	Log CRP					19.7	-5.72 to 45.0	2.92
	Log IL-6					-25.8	-65.8 to 14.2	2.04
			N = 96, $aR^2 < 0.01$		N = 96, $aR^2 = 0.02$		N = 77, $aR^2 = 0.07$	
STS	Age (year)	0.22	-0.56 to 1.01	0.36	-0.48 to 1.19	0.37	-0.48 to 1.26	0.98
	Height (cm)			0.27	-0.45 to 0.98	0.27	-0.44 to 0.97	0.71
	Smoking (current)			8.69	-2.43 to 19.8	2.32	-10.8 to 15.4	0.15
	LTPA (Low)					**20.6**	4.28 to 36.8	7.95
	Log CRP					**18.8**	5.29 to 32.3	9.61
	Log IL-6					**-20.8**	-41.3 to -0.30	5.11
			N = 96, $aR^2 < 0.01$		N = 96, $aR^2 = 0.01$		N = 77, $aR^2 = 0.13$	
Balance	Age (year)	**1.90**	0.53 to 3.27	**2.48**	1.09 to 3.87	**2.73**	1.20 to 4.27	14.14
	Height (cm)			**1.24**	0.04 to 2.43	**1.31**	0.06 to 2.57	4.88
	Smoking (current)			**25.9**	7.23 to 44.6	**29.0**	5.56 to 52.4	6.86
	LTPA (Low)					**15.4**	13.7 to 44.5	1.25
	Log CRP					-14.2	-38.4 to 9.93	1.56
	Log IL-6					15.6	-20.9 to 52.0	0.81
			N = 91, $aR^2 = 0.07$		N = 91, $aR^2 = 0.15$		N = 72, $aR^2 = 0.20$	

Significant β-coefficients are indicated in bold font when $p < 0.05$. Note that the dependent variables (DV) are 100 ln so that coefficients represent the symmetric percentage change in the DV per one unit change in the independent variables (IV). ΔR^2 indicates the squared semi-partial correlation coefficient for each IV.

CI, confidence interval; HGS, handgrip strength; LTPA, leisure-time physical activity; CRP, C-reactive protein; IL-6, interleukin-6; aR^2, adjusted coefficient of determination; FFM, fat-free mass; $a\dot{V}O_{2max}$, absolute maximal rate of oxygen uptake; $r\dot{V}O_{2max}$, relative maximal rate of oxygen uptake; FEV_1, forced expiratory volume after 1 s; FVC, forced vital capacity; STS, sit-to-stand test.
